# *Strongyloides stercoralis* is a cause of abdominal pain, diarrhea and urticaria in rural Cambodia

**DOI:** 10.1186/1756-0500-6-200

**Published:** 2013-05-20

**Authors:** Virak Khieu, Sophanaroth Srey, Fabian Schär, Sinuon Muth, Hanspeter Marti, Peter Odermatt

**Affiliations:** 1 National Centre for Parasitology, Entomology and Malaria Control, Ministry of Health, Phnom Penh, Cambodia; 2 Department of Epidemiology and Public Health, Swiss Tropical and Public Health Institute, P.O. Box, CH-4002, Basel, Switzerland; 3 University of Basel, Basel, Switzerland; 4 Medical and Diagnostics Department, Swiss Tropical and Public Health Institute, Basel, Switzerland

**Keywords:** *Strongyloides stercoralis*, Clinical symptoms, Ivermectin, Cambodia

## Abstract

**Background:**

We document clinical manifestations of 21 patients heavily infected with *S. stercoralis* (more than 250 larvae in a single Baermann test) from a community in rural Cambodia, both before and three weeks after ivermectin (200 μg/kg BW, single oral dose) treatment.

**Findings:**

Out of 21 patients, 20 (95.2%), 18 (85.7%) and 14 (66.7%) reported frequent abdominal pain, diarrhea and periods of sensation of itching, respectively, during the previous six months; epigastric (11, 55.0%) and peri-umbilical (13, 65.0%) pains were most frequent. Five patients (23.8%) reported having experienced urticaria the week preceding the examination. One patient suffered from extended urticaria. Three weeks after treatment, most symptoms had been almost entirely resolved.

**Conclusions:**

In rural communities of Cambodia, strongyloidiasis with high parasite load is endemic. It is associated with substantial symptoms and clinical signs, particularly abdominal pain, diarrhea and urticaria. Access to adequate diagnosis and treatment is a pressing issue that needs attention.

## Findings

Strongyloidiasis*,* an infection of an intestinal parasitic nematode, affects about 30–100 million people worldwide [1,2]. It is endemic in areas where sanitary conditions are poor and where the climate is warm and humid [3]. The clinical manifestations of strongyloidiasis vary greatly according to infection intensity and the immune-status of the patient. It is thought that more than 50% of all infections remain asymptomatic [4-6]. In Cambodia, a recent study showed that 24.4% and 49.3% of schoolchildren were infected with strongyloidiasis and hookworm, respectively [7]. Here, we report on the clinical manifestations of 21 strongyloidiasis patients from the rural province of Preah Vihear in northern Cambodia, with high numbers of *S. stercoralis* larvae in their feces.

In early 2010, in a community-based survey in Rovieng district (Preah Vihear province), stool examinations were conducted for individuals in randomly selected households. Two stool samples were obtained on two consecutive days from each person and examined with Baermann [8] and Koga agar plate culture (KAP) [9] techniques for the presence of *S. stercoralis* larvae, as well as with a single Kato-Katz slide [10] per stool sample for the detection of further helminth infections. A temporary laboratory was set-up in the local health facility to perform the stool examinations (Baermann, KAP culture and Kato-Katz methods). Patients with more than 250 larvae in one of the Baermann examinations were revisited and a detailed clinical assessment was performed. Patients were then treated with a single oral dose of ivermectin, 200 μg/kg BW. All patients were observed for one hour following treatment for the occurrence of adverse effects. Three weeks later, all patients were visited again and the clinical assessment was repeated [7,11]. All positive cases were re-treated according to the guidelines of the National Helminth Control Program of Cambodia [12]. The study was approved by the Ethics Committee of the Cantons of Baselstadt and Baselland (EKBB, #16/10, dated 1 February 2010), Switzerland, and the National Ethics Committee for Health Research (NECHR), Ministry of Health, Cambodia. We obtained written informed consent from a patient’s next of kin for the publication of the image in this manuscript. A copy of this written consent has been made available for review by the Editor-in-Chief of this journal.

Of the 273 participants from seven rural villages in Rovieng district (Preah Vihear province) who provided two stool samples, 86 (31.5%) tested positive for *S. stercoralis* larvae in either the Baermann or/and KAP culture. The median age of the participants was 23 years (range: 2 – 84); 49.1% were female. Fifty-five participants (20.1%) did not attend school, while 172 (63.0%) received primary school education. Ninety participants (33.0%) possessed a toilet at home, while 101 (37.0%) reported defecating in latrines. Most participants (96.3%) had shoes and wore them while defecating. Cows (79.8%), chickens (75.5%) and pigs (67.8%) were the most commonly reported domestic animals owned by the households.

Of the 86 *S. stercoralis* cases, 21 (24.4%) were found to have high intensity *S. stercoralis* infection. The median larvae count in the Baermann examination was 790 (range: 251–6849). The median age of the patients was 11 years (range: 5–67); 23.8% were females. Eleven patients (52.4%) were younger than 16 years. Eight patients (38.1%) had no schooling, 10 (47.6%) completed primary school, and three patients (14.3%) had attended a secondary school. Seven of the patients (33.3%) were additionally infected with hookworms, with a median number of 48 eggs per gram (range: 24 – 216) as assessed by the Kato-Katz technique.

Twenty (95.2%), 18 (85.7%) and 14 (66.7%) patients reported frequent abdominal pain, diarrhea and episodes of itching sensation (uticaria), respectively, during the previous six months (Table 1). Among reported abdominal pains, epigastric (11, 55.0%) and peri-umbilical (13, 65.0%) pains were most frequent. Diarrheal episodes were characterized by liquid and semi-liquid (17, 94.4%) and greasy (13, 72.2%) stools. Five patients (23.8%) reported the presence of urticaria during the week preceding the examination. In addition, 11 patients (52.4%) reported a cough lasting longer than a week in the previous six weeks. Other symptoms included tiredness (8, 38.1%); anorexia (6, 28.6%); and pale conjunctiva (8, 38.1%), which included three patients infected additionally with hookworms. Most symptoms (abdominal pain, diarrhea and itching) were almost entirely resolved in the three-week period following ivermectin treatment. Anorexia, abdominal pain, diarrhea and urticaria were reduced statistically significant (p < 0.05). One six-year old boy experienced vomiting within an hour after ivermectin treatment.

**Table 1:** **Clinical symptoms of patients with high intensity *Strongyloides stercoralis *infection: before and after ivermectin treatment, 2010**

**Clinical Examination**	**Before treatment**	**After treatment**	
		***S. stercoralis *negative**	***S. stercoralis *positive**
	**N = 21, n (%)**	**N = 18, n (%)**	**N = 3, n (%)**
Tired (yes)	8 (38.1)	1 (5.5)	1 (33.3)
Anorexia/loss of appetite (yes)	6 (28.6) *	0	0
Abdominal pain (yes)	20 (95.2) *	4 (22.2)	2 (66.7)
Epigastric pain	11 (55.0)	1 (25.0)	1 (50.0)
Right hypochondrial pain	6 (30.0)	1 (25.0)	0
Left hypochondrial pain	1 (5.0)	0	0
Peri-umbilical pain	13 (65.0)	3 (75.0)	2 (100)
Under umbilical pain	2 (10.0)	0	0
Diarrhea (yes)	18 (85.7) *	3 (16.6)	2 (66.7)
Greasy	13 (72.2)	2 (66.7)	1 (50.0)
Bloody	9 (50.0)	1 (33.3)	1 (50.0)
Liquid/Semi-liquid	17 (94.4)	3 (100)	2 (100)
Constipation more than a week (yes)	3 (14.3)	2 (11.1)	0
Urticaria on body, hands or legs during one to two weeks (yes)	14 (66.7) *	2 (11.1)	1 (33.3)
Urticaria on the body and hand during one week, particularly at night (yes)	5 (23.8)	0	1 (33.3)
Cough more than one week. Most cough with sputum and mostly at night (yes)	11 (52.4)	4 (22.2)	1 (33.3)
Pale conjunctiva (yes)	8 (38.1) *	0	0
Febrile at night during 3 days (yes)	1 (4.8)	0	0

* Significant difference between before and after treatment: p < 0.05 (McNemar test).

Three weeks after treatment, three patients (14.3%) were still *S. stercoralis* positive: a nine-year old boy had three *S. stercoralis* larvae in the Baermann test of the second stool and also a hookworm infection, revealed by the Kato-Katz (first stool sample negative); an eleven-year old girl had a few *S. stercoralis* larvae in the KAP culture of the second stool sample (first stool sample negative); and an eight-year old girl had 642 and 960 larvae in the Baermann tests, in addition to being infected by hookworms. Two of these patients reported abdominal pain and diarrhea and one patient reported experiencing cough and itching (Table 1). All three were retreated with ivermectin (200 μg/kg BW, single oral dose).

**Figure 1:**
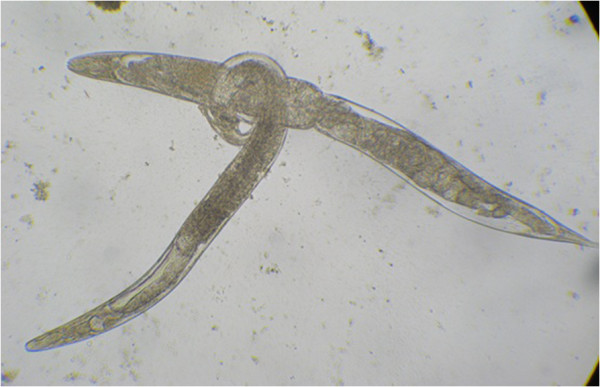
**Mating adult *Strongyloides stercoralis *observed on Koga agar plate culture.**

**Figure 2:**
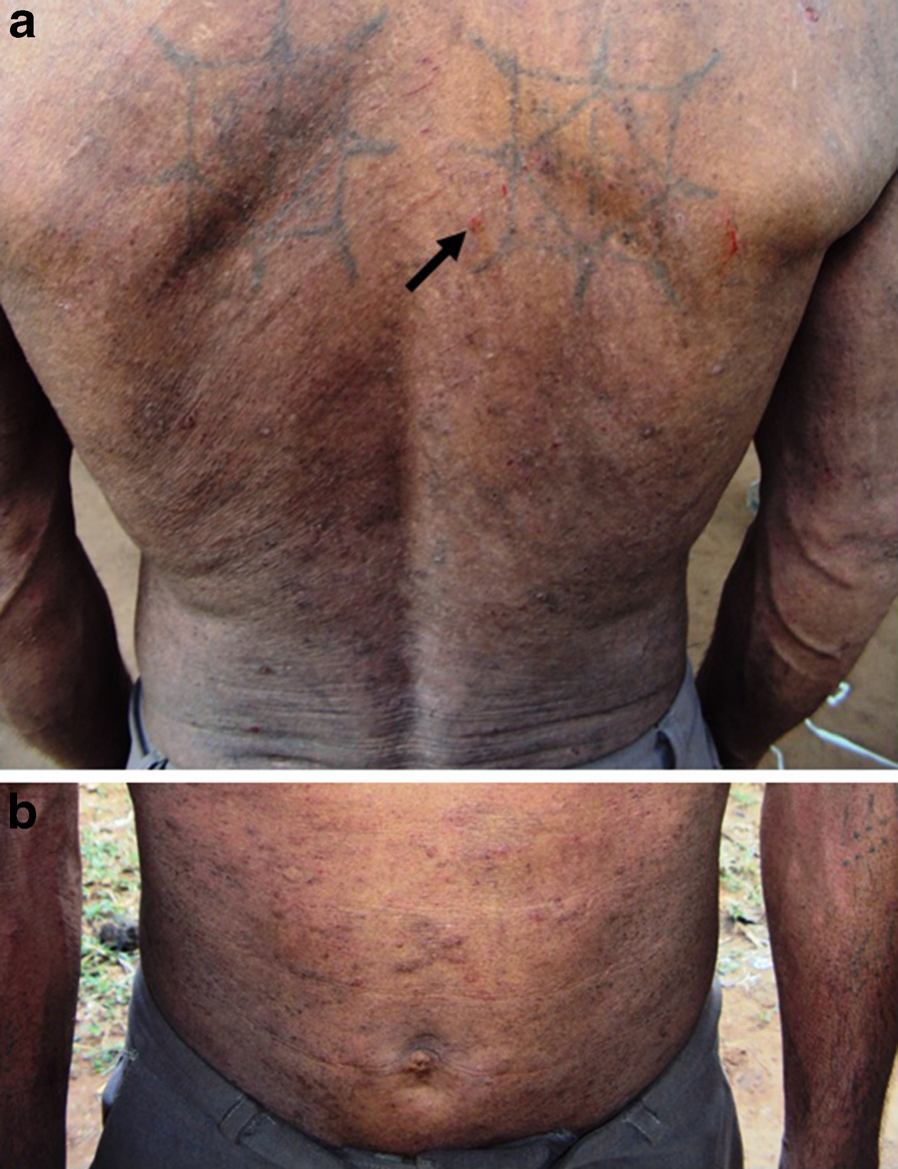
Extended dermatitis on back (a) and abdomen (b) in 43 years old farmer.

During a visit to the communities, a 43 year-old farmer, living in the rural eastern part of Preah Vihear province, was diagnosed with a *S. stercoralis* infection with 924 and 478 larvae present in his two Baermann examinations. Larvae and adult *S. stercoralis* were detected in KAP culture examinations of the stools (Figure 1). He was co-infected with hookworms. The patient presented with abdominal pain, diarrhea, nausea, vomiting, fever, and a pronounced and persistent skin rash, which had been present with extensive itching for more than two years. The rash was observed on the back, chest, abdomen and extremities (Figure 2a, b) and, due to frequent and intense scratching, showed signs of focal infection (Figure 2a, arrow). Three weeks after treatment with a single oral dose of ivermectin (200 μg/kg BW) and a single oral dose of mebendazole, the patient’s rash had almost disappeared and he was free of episodes of intensive itching.

Most of the patients had experienced at least one episode of abdominal pain, diarrhea and itchiness during the preceding six months. These symptoms had not been diagnosed or treated in this setting where poor and vulnerable people have only limited or even no access to health facilities. Additionally, in Cambodia, adequate diagnostic tests for strongyloidiasis are not available at public health facilities, including central level. Within the confines of our study, we could not diagnose additional underlying medical conditions such as gastritis, allergy or protozoan infections. However, it seems that other etiologies were unlikely to have played a major role in symptoms, as most symptoms dramatically resolved after ivermectin treatment.

Ivermectin, the drug of choice for strongyloidiasis [13], is not available in Cambodian health facilities, except for a few pharmacies in Phnom Penh. It is sold at USD 10.00 per tablet of 3 mg, which is not affordable for local people. Albendazole, the alternative drug for treatment of strongyloidiasis but with a lower efficacy, is recommended where ivermectin is not available [14], but the therapeutic regimen for strongyloidiasis is not established in the guidelines of the National Helminth Control Program of Cambodia [12].

This report of clinical manifestations in patients with high intensity of *S. stercoralis* infection, from communities in northern rural Cambodia, documents the severity of clinical symptoms associated with *S. stercoralis* in a population living in a poor setting with virtually no access to diagnosis and treatment. Certainly, many socio-economically and environmentally similar areas exist throughout Southeast Asia and elsewhere in resource poor countries.

## Competing interests

The authors declare that they have no competing interests.

## Authors’ contributions

VK carried out the field work, performed analysis and wrote the manuscript with PO. SS participated in the study design and field data collection. FS conducted the laboratory analysis. SM conceived the study and participated in design and coordination. HM participated in laboratory coordination. PO conceived and designed the study and interpreted data and wrote the manuscript together with VK. All authors contributed to manuscript revisions and approved the final manuscript.
